# Ambulatory chest drainage with advanced nurse practitioner-led follow-up facilitates early discharge after thoracic surgery

**DOI:** 10.1007/s11748-022-01873-9

**Published:** 2022-10-10

**Authors:** Oliver J. Harrison, Maria Elena Vilar Alvarez, Victoria Snow, Alessandro Tamburrini, Edwin Woo, Lukacs Veres, Martin H. Chamberlain, Aiman Alzetani, the drain clinic working group

**Affiliations:** 1 Department of Thoracic Surgery, University Hospital Southampton, Southampton, UK; 2 University of Southampton Medical School, University of Southampton, Southampton, UK; 3 Department of Thoracic Surgery, Mailpoint 46, D-Level North Wing, University Hospital Southampton, Tremona Road, Southampton, Hampshire SO17 1ST UK

**Keywords:** Ambulatory chest drain, Nurse-led clinic, Prolonged air leak, Excessive fluid leak, Enhanced recovery, Early discharge, Cost analysis

## Abstract

**Objectives:**

To demonstrate the safety and feasibility of advanced nurse practitioner-led (ANP-led) outpatient follow-up after discharge with ambulatory chest drains for prolonged air leak and excessive fluid drainage.

**Methods:**

Patients discharged with ambulatory chest drains between January 2017 and December 2019 were retrospectively reviewed. Discharge criteria included air leak < 200 ml/min or fluid drainage > 100 ml/24 h on a digital drain. Patients were reviewed weekly in the clinic by ANPs, a highly skilled cohort of nurses with physician support available. Outcomes included length of stay, duration of air or fluid leak and complications.

**Results:**

Two-hundred patients were included, amounting to 368 clinic episodes. The median age was 68 ± 13 years and 119 (60%) were male. 112 (56%) patients underwent anatomical lung resection (total anatomical lung resections during the study period = 917) equating to a discharge with ambulatory chest drain rate of 12.2% in this group. The median length of stay was 6 ± 3 days and 176 (88%) patients were discharged with air leak versus 24 (12%) with excessive fluid drainage. The median time to drain removal was 12 ± 11 days. Complications occurred in 16 patients (8%) and 12 (6%) required readmission. An estimated 2075 inpatient days were saved over the study period equating to an annual cost saving of £123,167 (US$149,032) per annum.

**Conclusions:**

Patients with air leak or excessive fluid drainage can safely be discharged with ambulatory chest drains, allowing them to return to their familiar home environment safely and quickly. ANP-led clinics are a robust and cost-effective follow-up strategy and are associated with a low complication rate.

## Introduction

Persistent air leak and prolonged fluid drainage are major factors contributing to extended length of hospital stay after thoracic surgery and represent a significant cost burden to healthcare institutions [1, 2]. Historically, when length of stay was measured in weeks rather than days, most air leaks settled before the patient was considered medically fit for discharge. Now, in the era of enhanced recovery, patients are ready for discharge far sooner, and the problem of persistent air leak has manifested.

Ambulatory chest drains allow patients to be discharged home with persistent air leak and fluid drainage, which effectively transfers the workload and cost of this complication to the outpatient service. While physician-led outpatient clinics are more cost-effective than prolonged inpatient care, such clinics are usually very busy and adding another cohort of patients may be challenging [3]. In our institution, advanced nurse practitioners (ANPs) have been an integral part of the thoracic surgery team for over a decade. More recently, these highly skilled nurses have established an ambulatory chest drain clinic with support from a thoracic surgeon on-site. Our perception is that ANP-led outpatient drain clinics are safe and highly cost-effective compared to traditional inpatient management, but there is a paucity of literature to support this belief. Therefore, the aim of this study was to elucidate and report the complications and cost-effectiveness of ANP-led ambulatory chest drain clinics at a large UK teaching hospital.

## Methods

### Patient selection and statistical analysis

**Fig. 1:**
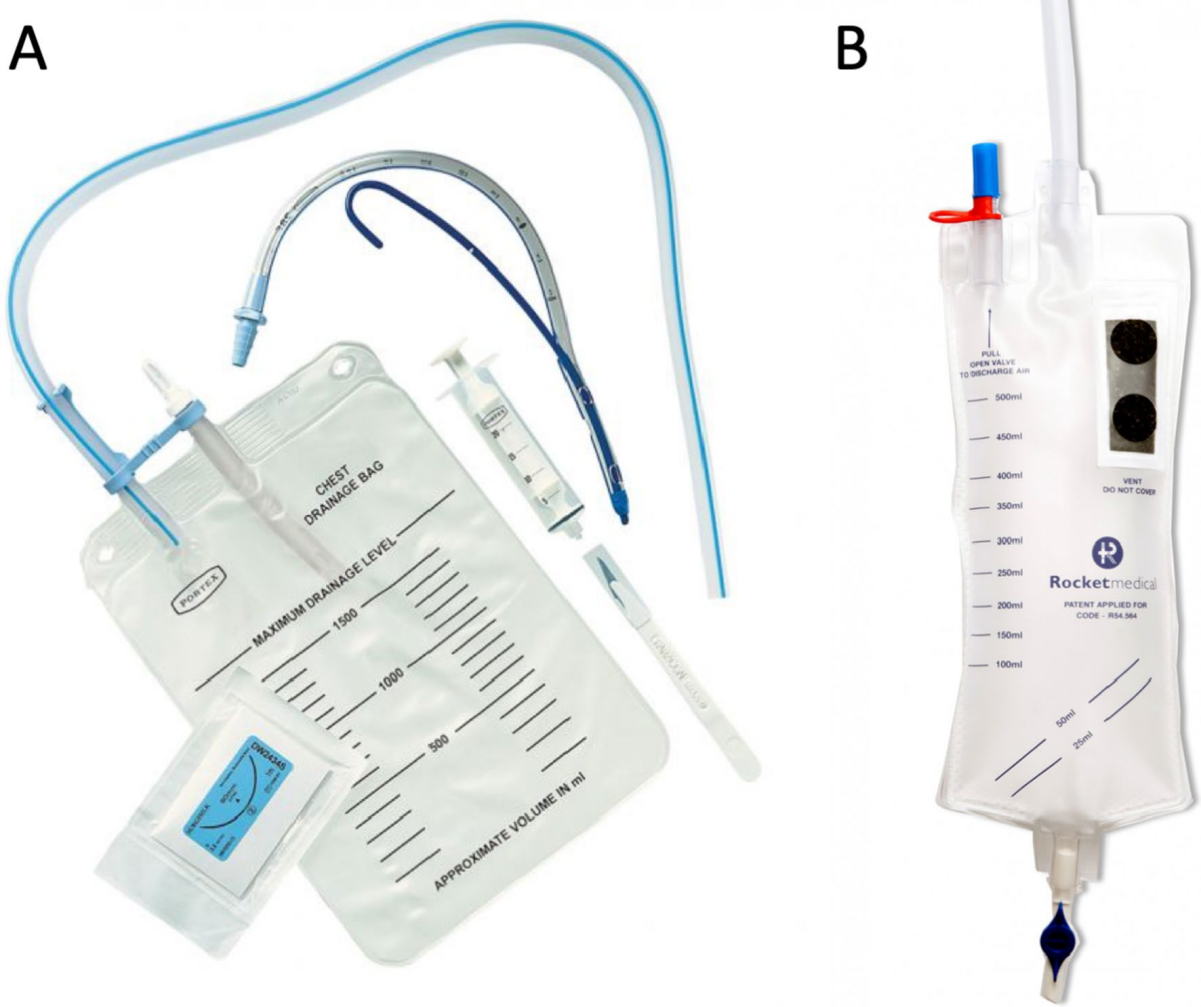
Examples of the ambulatory chest drains used in the present study. **A** Portex® Ambulatory Chest Drainage System (Smiths Medical, USA), **B** Rocket® Ambulatory Bag (Rocket Medical, UK)

Institutional review board (IRB) approval was obtained (ZAUD ID 6852) for retrospective data collection for all patients being discharged from our service with their chest tube attached to a ‘flutter bag’ or ‘rocket bag’ ambulatory chest drain (Smiths Medical, USA and Rocket Medical, UK respectively; Fig. 1) between January 2017 and December 2019. Given the retrospective nature of this work, the need for informed consent was waived by our institution. Procedures were performed by five surgeons in a large UK regional referral center. Our institution boasts of a well-established ANP-led chest drain clinic which was founded in 2016 and is run by a team of five ANPs. Data points included patient demographics, pulmonary function, operation performed (if applicable), length of stay, duration of clinic follow-up and any reported complications (readmission, infections including pneumonia or chest drain site, unmanageable pain and drain falling out or blocking). Current NHS England national pay scales were used to estimate nursing salary for the purpose of cost-effective analysis. Statistical analysis was performed with IBM SPSS Statistics (v26), and median values were given as ± interquartile range. Chi-squared test was used to test for differences in complication rates and *p* < 0.05 was taken as statistically significant. Bonferroni correction was employed for multiple comparisons.

### Criteria for discharge with an ambulatory chest drain

- **Patients with air leak**
  o Persistent air leak (< 200 ml/min on a Thopaz electronic drain system [Medela, UK] at − 0.8 kPa suction).
  o Absence of new clinical symptoms within 4 h of connecting an ambulatory chest drain.
  o Satisfactory lung expansion on chest X-ray after > 4 h on an ambulatory chest drain.
  o Established patient’s compliance with ambulatory chest drain management.
  o Community nurse able to review patient 48 hourly.
  o Patient medically fit for discharge.
- **Patients with excessive fluid**
  o Persistent fluid drainage following pleural effusion or empyema surgery > 100 ml/24 h.
  o Same criteria as above.

### Follow-up protocol

The ANP-led clinic runs twice per week on Tuesday and Thursday. One ANP is present and a thoracic surgeon is always available on-site for support if needed. Patients are reviewed on a weekly basis beginning 1-week post-discharge. Every clinic attendee undergoes a thorough assessment, including medical history, clinical examination and measurement of vital parameters (including blood pressure, heart rate, respiratory rate and oxygen saturation). Particular attention is given to the chest drain insertion site for signs of infection or any loosening of the stitch. The fluid contents of the bag are inspected and compared to the discharge observations. The flutter valve on the bag is inspected for patency and the patient is asked to cough to check for ongoing air leak. If air leak or excessive fluid drainage persists, the patient is re-booked to attend the clinic a week later. In the absence of air leak or on cessation of excessive fluid drainage, the drain is removed and following a satisfactory post-removal chest X-ray, the patient is discharged from the clinic.

## Results

### ANP-led clinic outcomes

The main study results are displayed in Table 1. Two-hundred patients were discharged with an ambulatory chest drain during the study period amounting to 368 ANP-led clinic episodes. The median age was 68 (range 25–89) years and 119 (60%) patients were male. 112 (56%) patients underwent anatomical lung resection (lobectomy, bilobectomy or segmentectomy). For comparison, a total of 917 patients underwent anatomical lung resection at our institution during the study period, equating to a discharge with an ambulatory chest drain rate of 12.2% in this group. The overall median postoperative/drain insertion length of stay was 6 (± 3) days and 176 (88%) patients were discharged with air leak versus 24 (12%) with excessive fluid drainage. None of the patients discharged with an ambulatory chest drain for excessive fluid drainage had undergone anatomical lung resection (23 pleural effusion and 1 empyema). There were no significant differences between outcomes for patients discharged with air leak versus excessive fluid drainage. Median time to drain removal following discharge was 12 (± 11) days and median number of clinic attendances was 2 (range 1–6). Complications occurred in 16 patients (8%). Ten (5%) patients developed a lower respiratory tract or chest drain insertion site infection, three (1.5%) patients reported unmanageable pain, two (1%) patient’s drains fell out and one (0.5%) patient’s drain was blocked. Of the 16 patients developing complications, 12 (6%) required readmission (Table 2). Median day post-discharge for readmission was 6 (± 5) days, median length of stay during readmission was 6 (± 4) days and all but three patients’ complications resulting in readmission were Clavien–Dindo class 2 or less. Discharge with ambulatory chest drain after pneumothorax surgery appeared to be associated with a higher frequency of complications (*p* = 0.009). However, after applying the Bonferroni correction (Bonferroni correction for multiple comparisons gives adjusted *p* = 0.004 as statistically significant), we could not demonstrate any significant relation between occurrence of complications and surgical procedure performed (Table 3). Assuming air leaks ceased (on average) midway between the penultimate and final outpatient clinic attended, approximately 2156 inpatient days were saved by use of ambulatory chest drains over the study period.

**Table 1:** Main study data

Age (years)	68 ± 13
Gender (*n*; M:F)	119:81
FEV1 (% predicted)^a^	84 ± 33
DLCO (% predicted)^a^	70 ± 28
Procedure performed (*n*; %)	
Lobectomy	94 (47%)
Wedge resection	25 (12.5%)
Pleural effusion	22 (11%)
Pneumothorax surgery	16 (8%)
Segmentectomy	9 (4.5%)
Bilobectomy	9 (4.5%)
Empyema debridement	6 (3%)
Trauma (pneumothorax)	4 (2%)
Lung volume reduction surgery	4 (2%)
Bedside pleurodesis	3 (1.5%)
Decortication	3 (1.5%)
Post-surgical readmission requiring drain	3 (1.5%)
Other	2 (1%)
Reason for clinic review (*n*; %)	
Air leak	176 (88%)
Excessive fluid	24 (12%)
Postoperative length of stay (days)	6 ± 3
Number of clinics attended (*n*)	2 ± 1
Time to post-discharge drain removal (days)	12 ± 11
Complications post-discharge (*n*; %)	
Total complications	16 (8%)
Required readmission	12 (6%)
Infection (LRTI or chest drain site)	10 (5%)
Pain	3 (1.5%)
Drain fell out	2 (1%)
Drain blocked	1 (0.5%)

Median ± interquartile range

*FEV1* forced expiratory volume in 1 s, *LRTI* lower respiratory tract infection, *DLCO* diffusion capacity of carbon monoxide

^a^Values for lobectomy, bilobectomy and segmentectomy only

**Table 2:** Patients requiring readmission *n* = 12

Patient ID	Days post-discharge readmitted	Reason for readmission and treatment given	Clavien–Dindo class	Length of readmission stay (days)
1	8	LRTI treated with antibiotics	2	4
23	7	LRTI treated with antibiotics, oxygen and physiotherapy	2	11
50	6	Drain fell out. Readmitted for reinsertion, developed type 1 respiratory failure requiring non-invasive ventilation	4a	16
58	8	Purulent discharge from drain site. Drain removed and stoma bag placed over the drain site	1	4
74	12	LRTI and purulent discharge from the chest drain. Treated with antibiotics and chest drain removed	2	18
131	3	Drain fell out. Readmitted for reinsertion, treated for LRTI with antibiotics	3a	6
142	2	Pain at the drain site. Analgesia optimized	1	1
164	2	LRTI treated with antibiotics and drain removed	2	5
177	6	Purulent discharge from the drain site. Treated with antibiotics and drain removed	2	6
186	5	Drain fell out. Drain reinserted, removed before discharge	3a	3
196	3	Pain at the drain site. Analgesia optimized	1	1
197	11	LRTI and pain. Treated with antibiotics and analgesia optimized	2	6

*LRTI* lower respiratory tract infection

**Table 3:** Complication frequency by operation

Operation	Number of patients with complications (%)	*p* value (non-corrected)^a^
Lobectomy	8 (8.5)	0.76
Wedge resection	1 (4)	0.42
Pleural effusion	1 (4.5)	0.55
Pneumothorax	4 (25)	0.01
Segmentectomy	0 (0)	0.37
Bilobectomy	1 (11)	0.69
Empyema	0 (0)	0.48
Trauma (pneumothorax)	1 (25)	0.19
Lung volume reduction surgery	0 (0)	0.55
Bedside pleurodesis	0 (0)	0.62
Decortication	0 (0)	0.62
Post-surgical readmission requiring drain	0 (0)	0.62
Other	0 (0)	0.69

Complications included readmission, infections including pneumonia or chest drain site, unmanageable pain and drain falling out or blocking. *p* values refer to differences in the proportion of complications in each operation group

^a^Bonferroni correction for multiple comparisons gives adjusted *p* = 0.004 as statistically significant

### Cost-effectiveness analysis

An average ward bed at our institution costs approximately £200 (US$242) per day. The cost of 2156 inpatient days saved over the study period minus 81 days required for the 12 patients that were readmitted equates to approximately £415,000 (US$502,150) or £138,333 (US$167,383) per annum. Associated costs of the providing the ANP-led clinic include the drain consumable cost of £10.50 (US$12.71) per unit, the ANP clinic salary of approximately £216 per week (£108 per day at £18/US$21.78 per hour) and the total cost of a 30 min review for every patient every 48 h by a community nurse (approximately £9,702/US$11,739). After deduction of these, we estimate a total saving of £369,502/US$447,097 (£123,167/US$149,032 per annum).

## Discussion

With the introduction of enhanced recovery after surgery pathways in thoracic surgery, rapid and safe discharge has become the standard of care [4]. Length of stay after major lung resection has been reported at less than 24 h [5]. Our ANP-led ambulatory chest drain outpatient clinic facilitates early discharge allowing patients to return safely to the comfort of their home environment, optimizing inpatient capacity and inviting a significant cost saving for our institution.

Persistent excess fluid drainage, but particularly persistent air leak, is a major factor predisposing to prolonged length of stay after thoracic surgery [6]. Factors predicting postoperative air leak are well established and include reduced forced expiratory volume in 1 s (FEV1), lung volume reduction surgery (LVRS), upper lobectomy or bilobectomy and reduced diffusion capacity [7]. At our institution, the risk of persistent air leak or excessive fluid drainage and potential need for discharge with an ambulatory chest drain is conveyed to the patient during the consent process so that patients are fully prepared should it be required. In the UK national audit, our institution demonstrated the shortest length of stay after major lung resection (median 4 days) [8]. Undoubtedly, this is in part due to our implementation of an ambulatory chest drain protocol utilizing an ANP-led clinic to manage persistent air leak or excessive fluid drainage in the outpatient setting. The majority of persistent air leaks are no longer an inpatient problem. After establishing this program in 2016, indications for implementing the protocol have expanded beyond lung resections to include pleural effusion, pneumothorax and empyema surgery, air leak following trauma and lung volume reduction surgery. The use of ambulatory chest drain in traumatic pneumothorax has not been previously reported.

Nurse-led clinics are long established in the UK and are proven to be safe, cost-effective and popular with patients [9, 10]. The ANP training pathway is rigorous and performance is continually evaluated. ANPs are generally from a senior nursing background (e.g., ward manager) with established leadership skills and extensive clinical experience. The higher academic degree of Master of Science (MSc) in Advanced Clinical Practice is awarded on completion of training which typically takes between 3 and 5 years. Training modules include history taking and physical examination, research methods and evidence-based practice, diagnostics and decision making, pharmacology and prescribing. Training consists of a combination of theoretical knowledge and applied clinical skills assessed by a consultant (attending) physician. The ANPs in our institution are well supported. Our ambulatory chest drain patients are reviewed in a dedicated thoracic surgery treatment room on the inpatient ward meaning a thoracic surgeon is always close by to offer advice and support if required, though in practice this is rarely needed.

Outpatient chest drain management of air leak with ambulatory drainage is not a new concept. A number of larger studies have documented the success of a number of devices in reducing length of stay in patients with persistent air leak secondary to a range of etiologies [11–14]. Limitations of the Heimlich valve system include its predisposition to blocking from fibrinous debris and potential for increased incidence of ascending infection being an ‘open system’ [15]. More recently, ‘closed system’ devices have been adopted, for example the Express Mini 500 (Atrium Medical Corp, USA) and the mini Sahara (Teleflex, USA), which appear to reduce these risks to a degree [16, 17]. Only one other smaller UK study has previously reported outcomes of a nurse-led chest drain clinic [18]. Tcherveniakov et al. demonstrated the safety and cost-effectiveness of a nurse-led drain clinic versus physician-led follow-up demonstrating a significant cost saving. Similar to others, they tolerated fluid drainage of up to 200 ml/24 h and any degree of air leak off suction so long as the lung was fully expanded on chest X-ray. By comparison, the present study was marginally more conservative with air leak and excessive fluid drainage thresholds. As a consequence, duration of chest drainage post-discharge was shorter (12 days versus 19 days) in the present study. Also, complication rate was lower in the present study (8 versus 18%), perhaps in part because residual air space/pneumothorax (not requiring intervention) was not included as a complication.

A common threshold reported in the literature for air leak which can be safely managed on an ambulatory chest drain is no worsening surgical emphysema or new/enlarging pneumothorax on chest X-ray while on an underwater seal [12, 16–19]. The introduction of digital drains has allowed quantification of air leak and delivery of mobile suction although their true benefit is disputed [20, 21]. Digital drainage is standard practice in our institution and our threshold for converting to an ambulatory chest drain is < 200 ml per minute of air leak on a suction of − 0.8 kPa (gravity). Conversely, consensus on upper threshold of excessive fluid drainage for drain removal is lacking. Thresholds as high as 500 ml/24 h following major lung resection have been proposed [22]. However, values between 300 and 400 ml/24 h may be better supported by the literature [23]. A recent randomized trial deemed a threshold of 300 ml/24 h optimal after minimally invasive lung resection [24]. Our threshold for excessive fluid production of > 100 ml/24 h may seem over-cautious by comparison. However, following pleural effusion and empyema surgery we tolerate a much lower production before a drain can be removed to prevent recurrence. This is borne out by the observation in our study of no recurrence in the 24 patients discharged with excessive fluid drainage after pleural effusion or empyema surgery.

Outpatient chest drain management is very safe and complications are infrequent and mostly minor. Incidence ranges from 3 to 26% with pain and infection (including superficial drain site, pneumonia and empyema), with or without a need for readmission, most commonly reported [3, 15, 17, 18, 25]. Cerfolio et al. advocate discharging patients with prophylactic antibiotics and this practice is certainly supported by Reinersman et al., who reported an empyema rate of 16.9% in their cohort [12, 15]. However, this is not common practice among published reports with others citing antibiotic complications and microbial resistance as reasons to avoid this practice. It seems a randomized trial is needed to elucidate their true benefit. In our practice, aside from patients with empyema (who are all discharged on a course of oral antibiotics), we do not routinely discharge patients with an ambulatory chest drain with prophylactic antibiotics and we did not see a worrying rate of infection complications in this series. However, we use ‘closed system’ ambulatory chest drains which may be less prone to ascending infection than ‘open systems’ such as Heimlich valves.

Patient safety when utilizing ambulatory chest drains is of paramount importance and some patients are understandably anxious about being discharged with a chest drain. Therefore, education and engagement with the patient and their family is vital. Our institution has established a robust support framework for managing patients with ambulatory chest drains in the outpatient setting. Prior to discharge, patients are given both practical and written guidance and only when both the patient and the medical team are satisfied with sufficient understanding is the patient discharged with an ambulatory chest drain. Patients are given a telephone number staffed 24 h in case of concern and each patient is reviewed on a 48 h basis at home or at their family practice by a community nurse. Moving into the future, our goal is to empower our patients further by teaching them to perform a daily air leak test and establish a daily clinic so that ambulatory chest drains can be removed as soon as air leak stops. Similarly, for patients less able or lacking confidence to do this, utilizing digital drains with wireless capability would allow a patient’s air leak status to be remotely monitored saving unnecessary return trips to hospital and facilitating prompt removal once the air leak stops.

This study had a number of limitations. We did not directly compare our cohort to a group of patients discharged without ambulatory chest drains as this data is not currently readily available at out institution. Therefore, we cannot comment on whether the complication rate differs from patients without an ambulatory chest drain. Secondly, we did not get formal patient feedback to confirm that patients are satisfied with our service, but this will be integrated into our pathway in the near future. Finally, our cost-effective analysis is based on estimated values and did not include the cost of any ED attendances, physician consultations, imaging or treatments (e.g., chest X-rays and antibiotics) that our service was not informed about. Therefore, the true cost benefits are likely to differ from that which we report. Similarly, given the diversity of healthcare systems around the world, we are unable to conclude that our protocol would provide a cost saving in every healthcare setting.

In conclusion, discharge of patients with persistent air leak and excessive fluid drainage secondary to a range of causes (including traumatic pneumothorax) with follow-up in a nurse-led clinic is safe and highly cost-effective. While the primary focus should always be prevention of air leak in the first instance, ambulatory chest drains remain an option to allow patients to return to their familiar home environment safely and quickly with an acceptable complication rate. We advocate the use of ‘closed system’ ambulatory chest drains and avoidance of routine prophylactic antibiotics. Finally, nurse-led follow-up empowers specialist nurses to make decisions regarding drain management and expands their critical role in the enhanced recovery after thoracic surgery program.
